# Altered Splicing of the BIN1 Muscle-Specific Exon in Humans and Dogs with Highly Progressive Centronuclear Myopathy

**DOI:** 10.1371/journal.pgen.1003430

**Published:** 2013-06-06

**Authors:** Johann Böhm, Nasim Vasli, Marie Maurer, Belinda Cowling, G. Diane Shelton, Wolfram Kress, Anne Toussaint, Ivana Prokic, Ulrike Schara, Thomas James Anderson, Joachim Weis, Laurent Tiret, Jocelyn Laporte

**Affiliations:** 1 IGBMC (Institut de Génétique et de Biologie Moléculaire et Cellulaire), Illkirch, France; 2 Inserm, U964, Illkirch, France; 3 CNRS, UMR7104, Illkirch, France; 4 Université de Strasbourg, Illkirch, France; 5 Collège de France, Chaire de Génétique Humaine, Illkirch, France; 6 Université Paris-Est Créteil, CNM project, Ecole Nationale Vétérinaire d'Alfort, Maisons-Alfort, France; 7 INRA, UMR955 de Génétique Fonctionnelle et Médicale, Maisons-Alfort, France; 8 Department of Pathology, University of California at San Diego, La Jolla, California, United States of America; 9 Department of Human Genetics, Julius-Maximilian University, Würzburg, Germany; 10 Department of Neuropediatrics, University of Essen, Essen, Germany; 11 Institute of Comparative Medicine, Division of Companion Animal Sciences, University of Glasgow Veterinary School, Glasgow, United Kingdom; 12 Institute of Neuropathology and JARA Brain Translational Medicine, RWTH Aachen University, Aachen, Germany; The Jackson Laboratory, United States of America

## Abstract

Amphiphysin 2, encoded by *BIN1*, is a key factor for membrane sensing and remodelling in different cell types. Homozygous *BIN1* mutations in ubiquitously expressed exons are associated with autosomal recessive centronuclear myopathy (CNM), a mildly progressive muscle disorder typically showing abnormal nuclear centralization on biopsies. In addition, misregulation of *BIN1* splicing partially accounts for the muscle defects in myotonic dystrophy (DM). However, the muscle-specific function of amphiphysin 2 and its pathogenicity in both muscle disorders are not well understood. In this study we identified and characterized the first mutation affecting the splicing of the muscle-specific *BIN1* exon 11 in a consanguineous family with rapidly progressive and ultimately fatal centronuclear myopathy. In parallel, we discovered a mutation in the same *BIN1* exon 11 acceptor splice site as the genetic cause of the canine Inherited Myopathy of Great Danes (IMGD). Analysis of RNA from patient muscle demonstrated complete skipping of exon 11 and *BIN1* constructs without exon 11 were unable to promote membrane tubulation in differentiated myotubes. Comparative immunofluorescence and ultrastructural analyses of patient and canine biopsies revealed common structural defects, emphasizing the importance of amphiphysin 2 in membrane remodelling and maintenance of the skeletal muscle triad. Our data demonstrate that the alteration of the muscle-specific function of amphiphysin 2 is a common pathomechanism for centronuclear myopathy, myotonic dystrophy, and IMGD. The IMGD dog is the first faithful model for human *BIN1*-related CNM and represents a mammalian model available for preclinical trials of potential therapies.

## Author Summary

The intracellular organization of muscle fibers relies on a complex membrane system important for muscle structural organization, maintenance, contraction, and resistance to stress. Amphiphysin 2, encoded by *BIN1*, plays a central role in membrane sensing and remodelling and is involved in intracellular membrane trafficking in different cell types. The ubiquitously expressed *BIN1*, altered in centronuclear myopathy (CNM) and myotonic dystrophy (DM), possesses a muscle-specific exon coding for a phosphoinositide binding domain. We identified splice mutations affecting the muscle-specific *BIN1* isoform in humans and dogs presenting a clinically and histopathologically comparable highly progressive centronuclear myopathy. Our functional and ultrastructural data emphasize the importance of amphiphysin 2 in membrane remodeling and suggest that the defective maintenance of the triad structure is a primary cause for the muscle weakness. The canine Inherited Myopathy of Great Danes is the first faithful mammalian model for investigating other potential pathological mechanisms underlying centronuclear myopathy and for testing therapeutic approaches.

## Introduction

Amphiphysin 2 is one of the key factors in muscular membrane remodeling. The gene, *BIN1*, has recently been associated with two different muscle disorders: centronuclear myopathy (CNM, MIM #255200) [1] and myotonic dystrophy (DM, MIM #160900 and #602668) [2]. However, the muscle-specific role of the ubiquitous protein amphiphysin 2 and the pathological mechanisms underlying the muscle disorders are not well understood. This is mainly due to the lack of faithful animal models.

Centronuclear myopathies are characterized by a generalized muscle weakness, atrophy, predominance of type I fibers, and aberrant positioning of nuclei and mitochondria [3]. The different genetic forms are not or are only moderately progressive. The X-linked and dominant CNM forms result from mutations in the phosphoinositide phosphatase myotubularin (*MTM1*) and the large GTPase dynamin 2 (*DNM2*), respectively [4], [5]. The autosomal recessive form (ARCNM) is caused by mutations in *BIN1*, probably involving a partial loss-of-function as the protein level was found to be normal in previously described patients [1]. Amphiphysin 2, encoded by *BIN1*, contains a N-terminal amphipathic helix, a BAR (Bin/Amphiphysin/Rvs) domain, able to sense and maintain membrane curvature, a Myc-binding domain and a SH3 domain, both implicated in protein-protein interactions [6], [7], [8]. There are at least 12 different isoforms, mainly differing by the presence or absence of a phosphoinositide-binding domain and a clathrin-binding domain encoded by exon 11 and exons 13–16, respectively [9], [10]. The clathrin-binding domain is present in the brain isoforms, while the phosphoinositide-binding (PI) domain is found almost exclusively in skeletal muscle isoforms [10], [11], [12]. Sequencing of cDNA demonstrated that all *BIN1* skeletal muscle isoforms contain exon 11 [12]. All ARCNM mutations described to date are in ubiquitously expressed exons [1], [13], [14], [15], raising the question about the molecular basis of the muscle-specificity of the disease. The BAR domain mutations strongly decreased the amphiphysin 2 membrane tubulating properties when expressed in cultured cells, while SH3 truncating mutations were shown to impair the binding and recruitment of dynamin 2 [1].

Mis-splicing of the *BIN1* muscle-specific exon 11 was reported in different forms of myotonic dystrophy (DM) [2]. DM is one of the most common muscular dystrophies in neonates and adults, and results from the expression of mutant RNAs with expanded CUG or CCUG repeats leading to the sequestration of splicing factors and subsequent defects in RNA splicing. Splicing alterations of the muscle chloride channel *CLCN1* are suggested to be responsible for the myotonia, whereas aberrant splicing of the insulin receptor *INSR* gene is thought to cause insulin resistance in DM patients. Complete or partial skipping of *BIN1* exon 11 in congenital and adult DM was shown to involve structural T-tubule abnormalities and subsequently muscle weakness [2]. However, there are numerous splicing defects in DM. It is therefore challenging to assess the exact contribution of *BIN1* exon 11 skipping to the DM phenotype, even though severe hypotonia, respiratory failure and histopathological features such as fiber hypotrophy and centrally located nuclei in the congenital forms of DM show intriguing similarities to CNM.

Amphiphysins are key regulators of membrane curvature and trafficking [16]. They can sense membrane curvature and presumably promote the curvature and fission of membranes [17]. Membrane binding occurs via BAR domain dimers, presenting a positively charged concave site that interacts with the negative membrane charges [17]. Amphiphysins also bind and recruit other regulators of endocytosis to sites of plasma membrane inward budding [18]. Amphiphysin 1 expression is restricted to neuronal tissues and the protein regulates synaptic vesicle recycling in the brain [19]. Amphiphysin 2 is highly expressed in adult striated muscle and its expression increases during muscle cell maturation [10], [11], [20], [21]. The polybasic residues encoded by *BIN1* exon 11 are required for amphiphysin 2-induced membrane tubulation when exogenously expressed in cultured cells [1], [22]. In skeletal muscle, amphiphysin 2 is localized at the T-tubules, which are deep sarcolemmal invaginations enabling excitation-contraction coupling [11], i.e. the process converting an electrical stimulus into mechanical muscle work. This specific localization, together with the membrane tubulation properties of the muscle-specific isoform containing the PI domain, called iso8 or M-amphiphysin, has led to the suggestion that amphiphysin 2 is implicated in T-tubule biogenesis [22]. This is sustained by defects in the localization of nascent T-tubule markers such as caveolin 3 following *BIN1* downregulation in cultured cells [23], and by the abnormal T-tubule structure seen in drosophila with null mutations in *amph*, the unique ortholog of mammalian amphiphysins 1 and 2 [24]. While faithful animal models were previously characterized for the *MTM1* and *DNM2* related CNM forms [25], the perinatal lethality of *Bin1*-null mice precludes the analysis of the role of amphiphysin 2 in skeletal muscle [26]. Therefore, critical questions concerning the muscle-specific function of amphiphysin 2 in mammals and the pathological mechanism of *BIN1*-related CNM remain unanswered. The lack of a faithful animal model for autosomal recessive centronuclear myopathy is a hurdle for a better comprehension of the pathological mechanisms and for the development of therapeutic approaches.

In this study, we identified and characterized the first human *BIN1* mutation affecting the muscle-specific PI domain. We also identified a novel spontaneous canine model reproducing the human pathology and allowing investigations on the physiological role of amphiphysin 2 in skeletal muscle after birth. Characterization of the dog model revealed an important role for amphiphysin 2 in triad structure, and we provide the evidence for a physiological function of the membrane-deforming properties of amphiphysin 2 and its alternative splicing-dependent activity. Our data support the hypothesis that the alteration of the muscle-specific function of amphiphysin 2 on membrane remodeling is a common pathomechanism underlying several canine and human myopathies.

## Results

### *BIN1* exon 11 splice mutation in patients with rapidly progressive centronuclear myopathy

**Figure 1:**
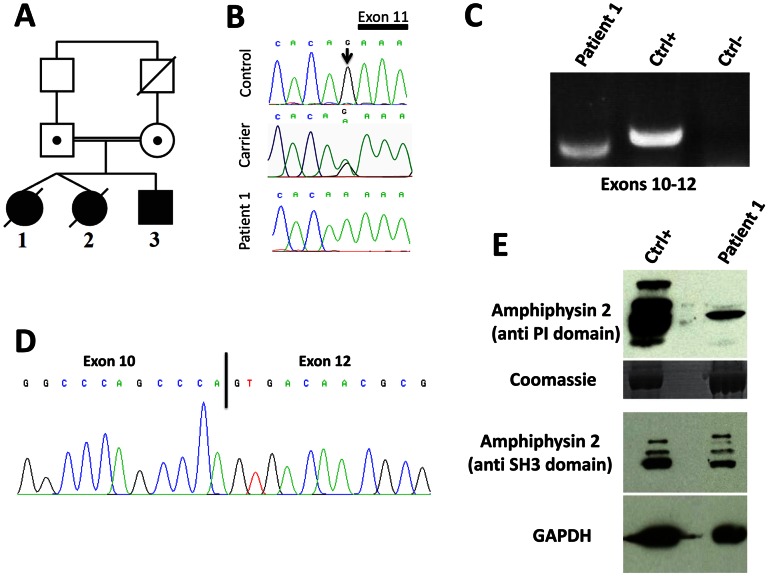
Human *BIN1* mutation of the exon 11 acceptor splice site and impact on splicing. (A) Pedigree and (B) Chromatopherogram. Patients 1 and 3 are homozygous for the IVS10-1G>A mutation, while both parents are heterozygous carriers. DNA from patient 2 was not available. (C) RT-PCR on mRNA isolated from muscle using primers encompassing *BIN1* exons 10–12 demonstrated amplification of a shorter product in patient 1 compared to a healthy control. For the negative control (Ctrl-) PCR was performed without cDNA. (D) Sequencing of the *BIN1* cDNA from muscle demonstrated skipping of *BIN1* exon 11 in patient 1. (E) Western blot analysis of patient muscle extracts detected a strong reduction of the amphiphysin 2 isoforms containing the exon 11 encoded PI-binding domain. The level of amphiphysin 2 detected with an anti-SH3 antibody was comparable between patient 1 and control.

To identify *BIN1* mutations affecting its function in skeletal muscle, we sequenced the muscle-specific exon 11 and the adjacent splice-relevant intronic regions in a cohort of 84 patients with various forms of centronuclear myopathy and without mutations in *MTM1*, *DNM2*, or in the other *BIN1* exons. We identified a homozygous *BIN1* exon 11 splice acceptor mutation (IVS10-1G>A) in two affected members from a consanguineous family from Turkey (Figure 1A and 1B). DNA was not available for the third affected member, who is expected to carry the same homozygous *BIN1* mutation as her monozygotic twin sister. The parents are healthy and do not present clinical signs of a muscle disorder. They are first-degree cousins and were found to be heterozygous for the *BIN1* exon 11 splice acceptor mutation, confirming autosomal recessive inheritance of the disease. The mutation was not found in unaffected individuals from different origins, including 37 DNAs from an ethnically matched control population, and is not listed in the SNP databases as dbSNP, 1000 genomes, or the NHLBI exome variant server.

Patients 1 and 2 are dizygotic twins. Pregnancy and birth, as well as motor and speech development were normal. General muscle weakness was diagnosed at 3.5 years. Hypotonia, muscle weakness (predominantly of the lower limbs), respiratory distress (VC 50%) and loss of motor skills were rapidly progressive and the twins died from acute pneumonia involving cardiac failure at age 5 and 7, respectively. Patient 3 is the younger brother, and as for his sisters, pregnancy, birth, motor and speech development were normal. Age of onset was 3.5 years and the myopathy was highly progressive, contrasting the slow progression of muscle weakness in the reported CNM cases with *BIN1* mutations in ubiquitous exons [1], [13], [14], [15]. Patient 3 presented with predominant proximal muscle weakness of the lower limbs requiring a wheelchair since the age of 5 years, facial weakness, but no respiratory distress. Eye movement defects, as seen in the majority of the *MTM1*, *DNM2* and *BIN1* patients, were not noted. Deep tendon reflexes were absent and the patient had progressive contractures in knees and ankles. Electrophysiological evaluation was normal or showed only unspecific myopathic changes, with normal nerve conduction velocity. Cardiac defects were not noted and CK levels were normal. Patient 3 is now 9 years old and presented at his last medical exam in April 2012 with low MRC grades for both upper and lower limbs.

### Impact of the human *BIN1* mutation on splicing

The *BIN1* IVS10-1G>A variation changes the AG acceptor splice site into AA, and is predicted to impair exon 11 splicing by various algorithms. The wild-type acceptor site was recognized by NNSPLICE (score 0.84) and Human Splice Finder (88.5), while no acceptor splice site was predicted in the mutated sequence. To confirm an impact on exon 11 splicing, we performed RT-PCR after RNA isolation from a muscle biopsy of patient 1, amplified a fragment encompassing exons 10 to 12, and obtained a shorter product compared to the control (Figure 1C). To analyze the transcript(s), we cloned the PCR-products and sequenced the resulting clones. As we and others previously reported, the skeletal muscle *BIN1* isoforms contain exon 11, but lack exons 7 and 13 to 16. Exon 17 can be either present or absent, corresponding to isoform 8 or M-amphiphysin [10], [11], [12]. Among the 30 analyzed clones, only a single clone contained exon 11. Twenty-nine clones did not contain exon 11 and directly combined exon 10 with exon 12, demonstrating a major skipping of the in-frame exon 11 in the patient muscle (Figure 1D). The impact of the mutation on the amphiphysin 2 protein level in skeletal muscle was investigated by Western blot (Figure 1E). Using an anti-PI domain antibody, we detected several bands in the control as previously reported [1], most probably reflecting post-translational modifications of the different isoforms containing exon 11. In the patient muscle, we found a significant decrease of the level of the amphiphysin 2 isoform containing the PI domain, confirming exon 11 skipping in most *BIN1* muscle transcripts. The amphiphysin levels detected with the anti-SH3 domain antibody were similar in patient 1 and control. Together with the genetic data, we conclude that the rapidly progressive CNM form results from a splice mutation involving the skipping of the muscle-specific exon 11.

### *BIN1* exon 11 is required for membrane tubulation in muscle cells

**Figure 2:**
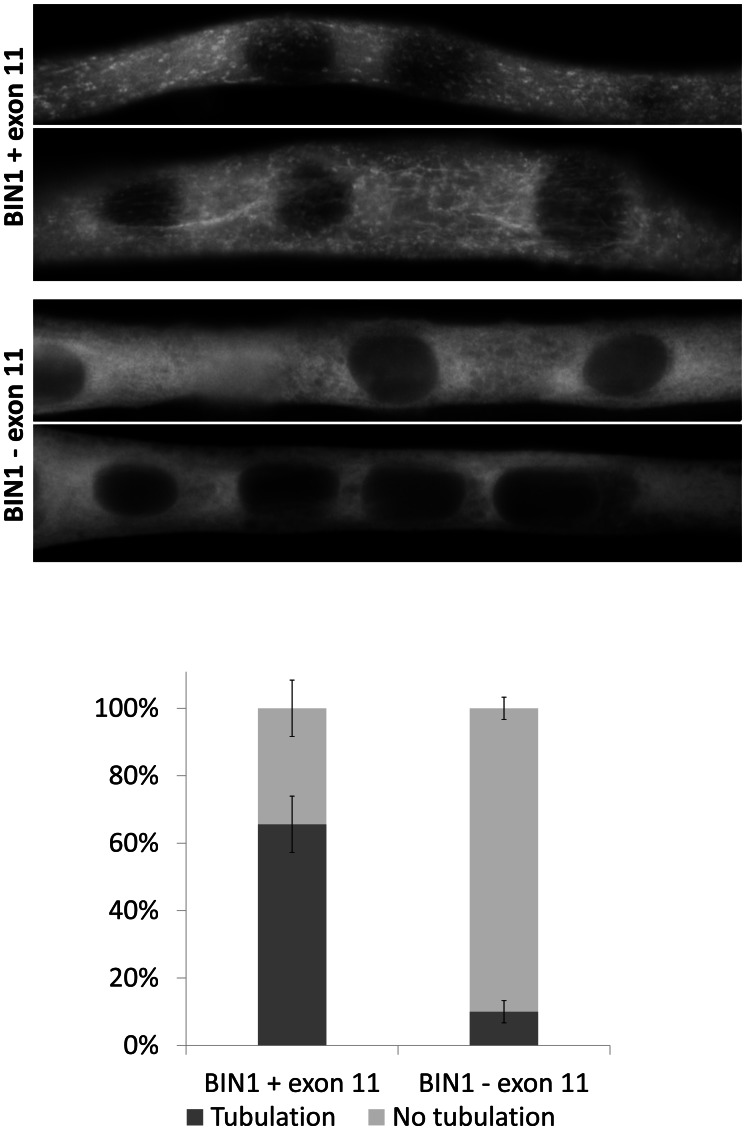
Essential role of *BIN1* exon 11 in membrane tubulation in myotubes. C2C12 myotubes overexpressing *BIN1* isoform 8 (including exon 11) showed strong tubulation, whereas *BIN1* isoform 9 (without exon 11) does not induce membrane tubulation 5 days post differentiation. Below: quantification of three independent experiments (>30 myotubes each) demonstrated that these findings were significant (p<0.01).

Previous publications demonstrated the importance of the amphiphysin 2 PI domain in PtdIns(4,5)P2 binding and membrane tubulation [1], . We transfected C2C12 cells with *BIN1* constructs including or excluding exon 11, and we induced the differentiation of the murine myoblasts into myotubes. Myotubes expressing the exon 11 containing isoform showed tubulation [22], [27], whereas the isoform without exon 11 did not induce this effect (Figure 2). Quantification revealed statistical significance. Immunolabelling of actin, caveolin-3 and RYR1 did not reveal obvious differences between the differentially transfected myotubes (data not shown), suggesting that the amphiphysin 2 PI domain is important for late muscle development or maintenance, rather than for early muscle development. This hypothesis is supported by the fact that the patients were unaffected at birth and during early childhood.

### *BIN1* exon 11 splice mutation causes the canine Inherited Myopathy of Great Danes (IMGD)

**Figure 3:**
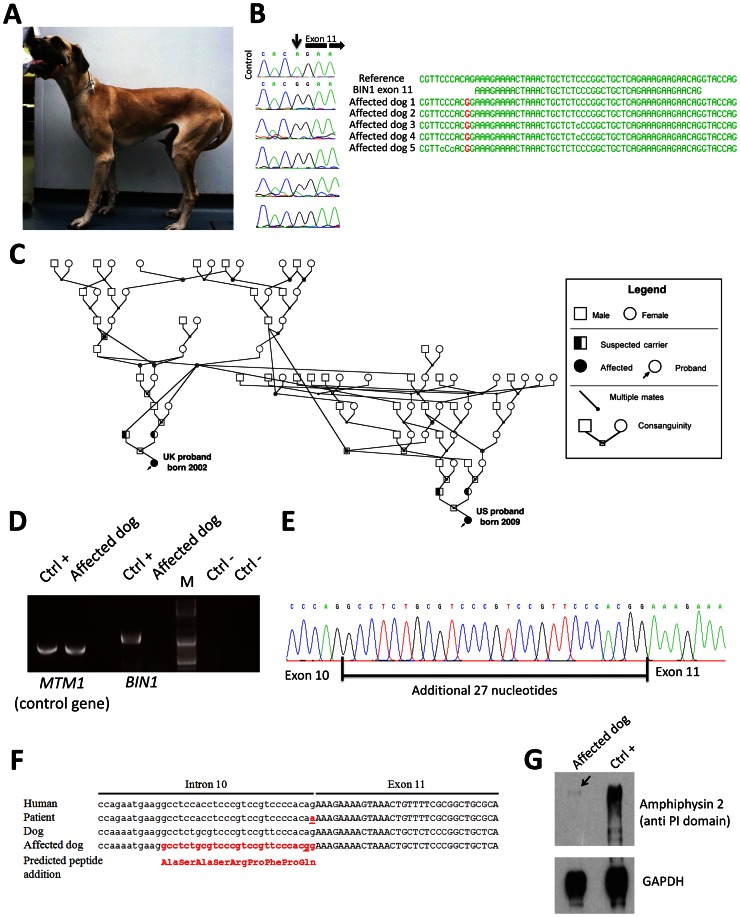
The canine Inherited Myopathy of Great Danes results from a *BIN1* mutation in the exon 11 acceptor splice site. (A) Picture of an affected 3-year-old Great Dane dog. (B) Chromatopherograms and sequence alignment showing the *BIN1* IVS10-2A>G mutation in 5 affected dogs. (C) Pedigree showing the distant relationship of two affected Great Dane dogs from the UK and US. (D) RT-PCR on skeletal muscle RNA showed a strong reduction of the *BIN1* RNA level in the IMGD dog compared to the healthy canine control. Amplification of a control gene (*MTM1*) was normal. M = Marker (E) Sequencing of the residual cDNA revealed the presence of 27 additional nucleotides due to the use of a weak cryptic 5′ splice acceptor site. (F) Sequence alignment of human and canine *BIN1* intron/exon boundary of exon 11. (G) Western blot using an anti-PI domain antibody showed a strong decrease of the amphiphysin 2 protein level.

The perinatal lethality of *Bin1*-null mice precludes investigations on the role of amphiphysin 2 in skeletal muscle maintenance [26]. To identify and characterize an animal model for *BIN1*-related CNM, we analyzed canine pedigrees with molecularly unsolved myopathies. The canine Inherited Myopathy of Great Danes (IMGD) is characterized by rapidly progressive muscle atrophy and exercise intolerance with an age of onset of about 6 months. Histological examinations of muscle biopsies from autosomal recessive cases from the UK, Canada and Australia revealed increased nuclear internalization and centralization [28], [29], [30], consistent with centronuclear myopathy. We excluded mutations in *MTM1* [31] and *PTPLA* [32] before sequencing the coding regions and intron/exon boundaries of the canine *BIN1* gene (XM_540990.3). We identified a homozygous AG to GG substitution of the *BIN1* exon 11 acceptor splice site in five dogs from Canada, US and UK (IVS10-2A>G; Figures 3A and 3B). CK values for the dogs were normal or slightly elevated. Pedigree reconstruction revealed a distant relationship between the US and one UK dog (Figure 3C) and a previous publication reported a common ancestor for all IMGD dogs in the UK [29]. The *BIN1* IVS10-2A>G mutation was not found in 112 healthy Great Danes and in 35 dogs from 12 other breeds, strongly suggesting its pathogenicity.

### Impact of the canine *BIN1* mutation on exon 11 splicing

Like the human *BIN1* IVS10-1G>A mutation, the canine *BIN1* IVS10-2A>G variation affects the exon 11 acceptor splice site. To assess its impact on splicing, we performed RT-PCR on RNA isolated from skeletal muscle biopsies and found a strong reduction of the *BIN1* RNA level compared to healthy controls and compared to a control gene (*MTM1*, Figure 3D). We however detected a faint signal of expected size and cloned the amplicon. All three clones contained exon 11 with 27 additional upstream nucleotides, encoding the amino acid sequence ASASRPFPQ (Figure 3E). This in-frame extension results from the disposition of a weak cryptic 5′ acceptor site. The intronic sequence upstream of exon 11 slightly differs between human and dog, possibly explaining the cryptic splicing in dogs versus exon skipping in human patients (Figure 3F). To confirm the impact of the splice mutation on the amphiphysin 2 protein level, canine muscle extracts were probed with an anti-PI domain antibody on Western blot. Compared to the healthy control, amphiphysin 2 was significantly reduced in the affected dog (Figure 3G). Using an anti-SH3 antibody we detected a strong reduction of all skeletal muscle amphiphysin isoforms (Figure S1) in accordance with the RT-PCR data. We conclude that the canine Inherited Myopathy of Great Danes results from a *BIN1* exon 11 splice mutation, provoking a strong reduction of the exon 11/PI domain-containing RNA and protein.

### Similar histopathology in affected humans and dogs

**Figure 4:**
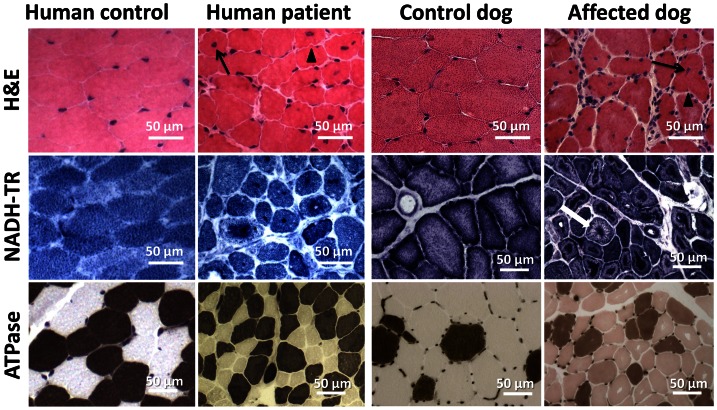
Histopathological comparison of muscles from human patient and IMGD dog. Human and canine muscle biopsy sections revealed nuclear centralization (arrows), fiber atrophy and lobulation as well as sarcolemmal invaginations (arrowheads) on H&E staining. NADH-TR staining demonstrated central dense areas in many fibers and “spoke of wheel” appearance in a few fibers (white arrow). ATPase staining (pH 4.3) revealed no or only a slight predominance of type I fibers compared to the age-matched controls.

Vastus lateralis muscle biopsies were performed for patient 1 as well as for patient 3 at the age of 3.5 years. H&E staining revealed prominent nuclear centralization (>60%, arrow), fiber atrophy and endomysial fibrosis (Figure 4), consistent with centronuclear myopathy. Similarly, H&E staining of biceps femoris muscle biopsies from affected dogs revealed nuclear internalization (>40%) and fiber atrophy. The central areas devoid of staining reflect perinuclear regions lacking myofibrils. Of note, the transverse muscle sections of patients and affected dogs showed an unusual lobulated appearance with indentations of the sarcolemma (arrowheads). NADH staining of human and canine sections revealed dense central areas in most fibers and “spoke of wheel” appearance in 5% of the fibers. ATPase staining showed no or only a slight predominance of type I muscle fibers as compared to the age matched controls. Gomori trichrome staining did not reveal any further abnormalities (data not shown). Taken together, human and canine histopathologies were comparable.

### Common ultrastructural and membrane defects in affected patients and dogs

**Figure 5:**
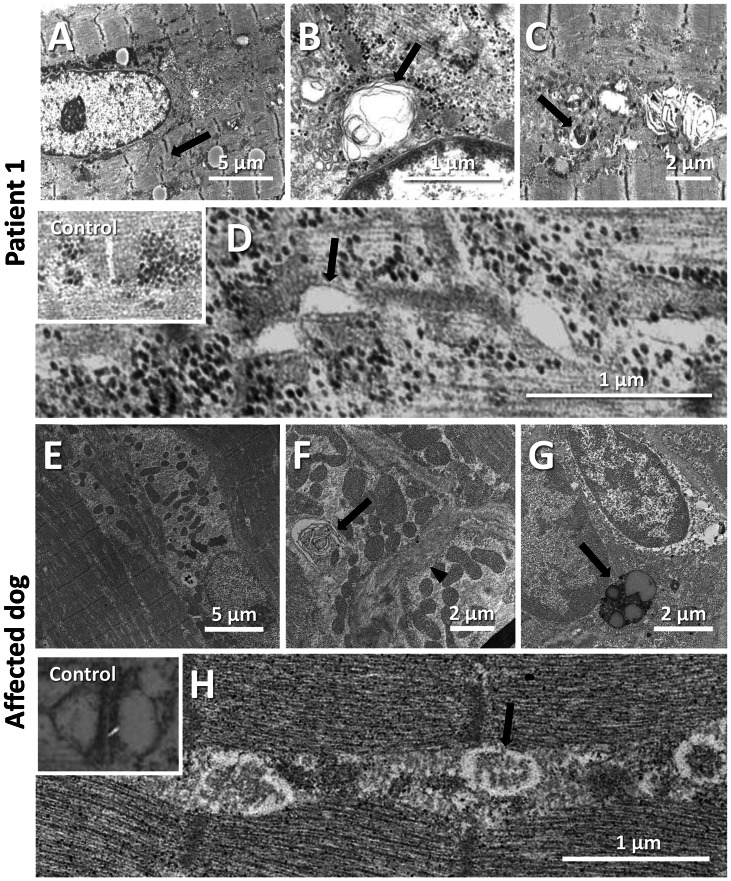
Common ultrastructural and membrane defects in affected human and dog. (A–D) Electron microscopic analysis of a patient biopsy showing a centralized nucleus surrounded by organelles and mild sarcomeric disarray (arrow, A), accumulations of membranes and vacuoles containing whorled membranes (arrow, B), autophagic vacuoles containing myelin-like material (arrow, C), and widened tubules at the triads (arrow, D). The inset shows normal triads in an age-matched biopsy. (E–H) Ultrastructural analysis of a IMGD dog biopsy revealed central nuclei surrounded by mitochondrial accumulations (E), membranous whorls (arrow, F), deep membrane invaginations (arrowhead, F), lipofuscin granules (arrow, G), and abnormal triads (arrow, H). The inserted picture shows a normal triad in an age-matched canine control.

To uncover the pathological defects underlying this highly progressive form of centronuclear myopathy and to validate the canine model, we analyzed human and dog muscle biopsies by electron microscopy. Ultrastructural analysis of the human muscle biopsy revealed centralized nuclei surrounded by an area devoid of myofibrils and containing glycogen granules and other organelles (Figure 5A, Figure S2), as commonly seen in *MTM1*, *DNM2* and *BIN1*-related CNM. Myofibrillar disintegration with occasional Z-band streaming (arrow, Figure 5A) was seen in the adjacent sarcomeres. Triad structures were found to be aberrant and we observed frequent enlarged structures, most probably originating from the sarcoplasmic reticulum (arrow, Figure 5D). We also noted other membrane alterations, including accumulations of membranes and tubules, vacuoles containing whorled membranes (arrow, Figure 5B), as well as a high number of myelin-like membranous structures suggestive of autophagosomes (arrow, Figure 5C). Likewise, ultrastructural analysis of muscle biopsies from an affected Great Dane dog showed nuclear internalization, mitochondrial accumulations around the internalized nuclei and myofibrillar disarray (Figure 5E, Figure S3). We furthermore found membranous whorls (arrow, Figure 5F) as reported for the X-linked CNM Labrador retriever model with *MTM1* mutation [31], deep membrane invaginations (arrowhead, Figure 5F), lipofuscin granules (arrow, Figure 5G), and abnormal triads in almost all fibers (arrow, Figure 5H). Sarcolemmal invaginations contained basement membranes and often pointed towards centralized nuclei. Taken together and considering the histological analysis described above, histopathology of IMGD dogs and human patients appear strikingly similar, emphasizing common alterations of membrane structures.

### Amphiphysin 2 is present but altered in affected human and dog muscles

**Figure 6:**
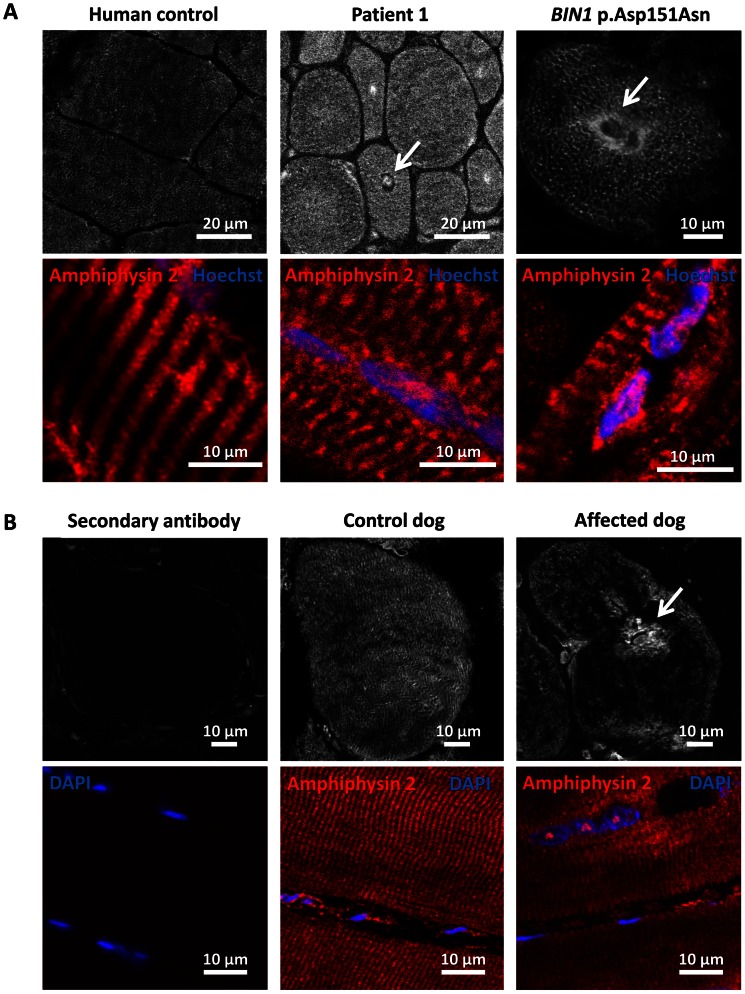
Amphiphysin 2 is present but altered in muscles from affected humans and dogs. (A) Amphiphysin 2 localization in control (left), patient 1 (middle) and a CNM patient with the p.Asp151Asn mutation (right). Arrows indicate abnormal accumulations of amphiphysin 2 around centralized nuclei in both patient muscles. (B) Abnormal localization of amphiphysin 2 on transversal and longitudinal muscle sections from an IMGD dog compared to a control. The secondary without the primary antibody was applied on control canine sections to withdraw non-specific background staining.

To further characterize the pathophysiology of the rapidly progressive human CNM and canine IMGD, we performed immunolocalization experiments on muscle biopsies. Using the R3062 antibody recognizing most amphiphysin isoforms or the PI-domain specific R2405 antibody, signals were detected as an intracellular network in transverse sections of human and canine controls (Figure 6). Signals were also detected in sections of muscles from patient and affected dog, reflecting the presence of different amphiphysin 2 isoforms as shown by Western blot. Despite the decrease of *BIN1* RNA in affected dogs, the remaining mis-spliced in-frame transcripts can explain the detection of amphiphysin 2 on muscle sections, especially because immunohistochemistry is not quantitative. The amphiphysin 2 network appeared however abnormal in patient and IMGD sections. In some fibers we noted central areas without any signal, while in other fibers accumulations around centralized nuclei were observed (arrows). To determine whether these anomalies were specific for the *BIN1* exon 11 splice mutation or rather a general *BIN1*-related CNM feature, we analyzed a muscle biopsy from a patient with the previously reported *BIN1* p.Asp151Asn mutation and a classical ARCNM phenotype [1]. We observed similar accumulations of amphiphysin 2 (Figure 6A), suggesting that different *BIN1* mutations in humans and dogs lead to similar amphiphysin 2 mis-localization in muscle.

### Alteration of triad and membrane trafficking regulators

**Figure 7:**
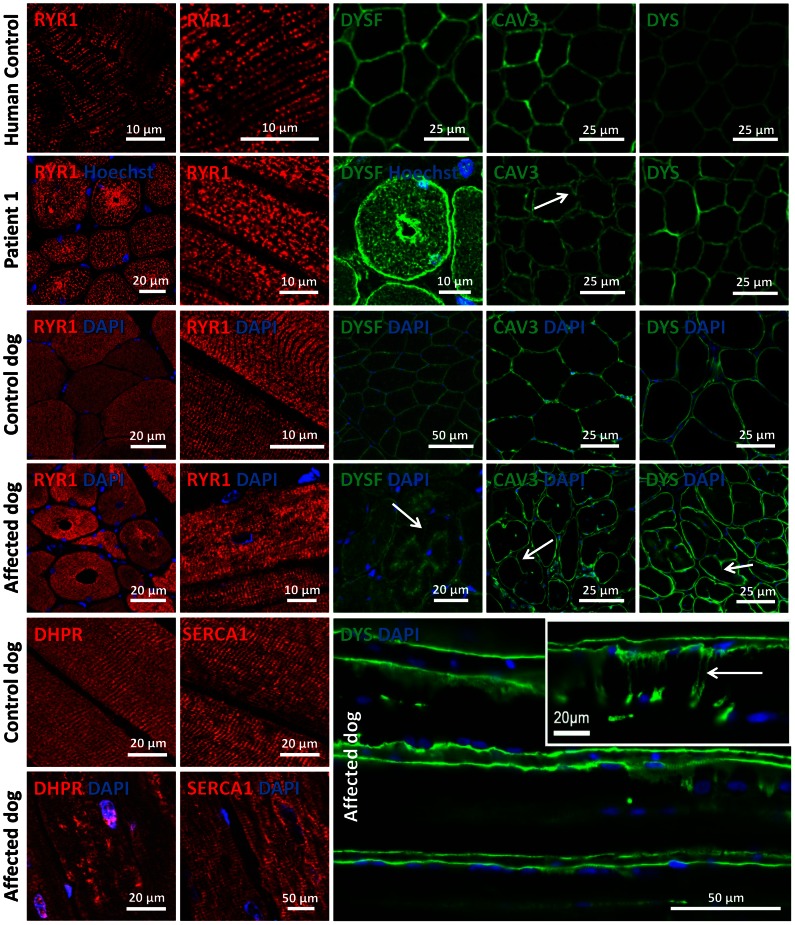
Alteration of triad components and proteins regulating membrane trafficking. Immunolocalization on longitudinal and transversal sections from patients and affected dogs revealed an abnormal pattern of the junctional sarcoplasmic calcium channel RYR1, the T-tubule marker DHPR and the longitudinal sarcoplasmic calcium pump SERCA1. Especially RYR1 was found to accumulate around internalized nuclei. Intracellular dysferlin signals were detected in patients and affected dogs, but not in the age-matched controls. Labeling of the sarcolemmal markers caveolin 3 and dystrophin demonstrated prominent lobulation and deep indentations of the plasma membrane in patients and affected dogs on transversal and longitudinal sections (arrows).

Amphiphysin 2 has been proposed to be implicated in T-tubule biogenesis, but the exact link has barely been documented in mammalian skeletal muscle [22]. We therefore examined the skeletal muscle triad using antibodies against the junctional sarcoplasmic calcium channel RYR1 and the T-tubule marker DHPR in human and dog (Figure 7). Both proteins were profoundly altered, showing focal accumulations or central areas without signal in the fibers. Compared to the control longitudinal sections, the transversal orientation of RYR1-labeled triads was lost in patient and canine muscle. Similarly, the longitudinal sarcoplasmic calcium pump SERCA1 was mislocalized in sections from affected dogs.

We next wanted to know whether the aberrant triad structure was concurrent with more generalized membrane defects. Dysferlin and caveolin 3, key regulators of membrane repair and trafficking [33], [34], were found to be mainly located at the sarcolemma in control muscle sections. In contrast, transverse sections of patient 1 and of an affected Great Dane dog revealed striking intracellular accumulations of dysferlin, mainly around central nuclei (Figure 7). Labeling of the sarcolemmal markers dysferlin, caveolin 3 and dystrophin confirmed the presence of numerous fibers with unusual lobulated and indented sarcolemma, representing deep sarcolemmal invaginations pointing towards the center of the fibers (arrows, Figure 7). Taken together, our data correlate the highly progressive human CNM and canine IMGD with general membrane alterations at the triad, the sarcolemma and within the fibers. However, these defects did not reflect a general disorganization of the sarcomere, as alpha-actinin labeling appeared largely normal (not shown). Staining of developmental myosin revealed no difference between affected and control dogs, indicating that there is no excessive fiber regeneration in IMGD dogs (Figure S3).

### Altered myotubularin localization in BIN1-mutated canine muscles

**Figure 8:**
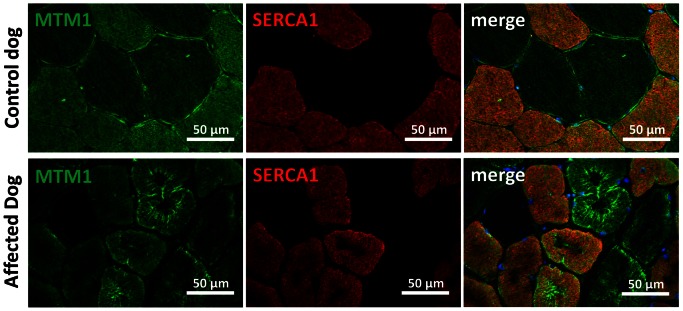
Myotubularin is mis-localized in IMGD muscle. In muscle sections from control dogs, myotubularin is predominantly expressed in type II fibers expressing SERCA1. In affected IMGD dogs, massive myotubularin accumulations formed a concentric network around the fiber center.

As *MTM1* is mutated in X-linked human and canine CNM, we investigated the localization of myotubularin in muscle sections of IMGD dogs. Myotubularin formed an intracellular network in control sections and the signal was stronger in type II fibers labeled with the SERCA1 antibody (Figure 8). In both analyzed IMGD muscles, myotubularin was mainly located as concentric strands pointing to the center in both type I and type II fibers. We conclude that altered splicing of *BIN1* has a strong impact on myotubularin localization in muscle, revealing a potential link between IMGD and X-linked CNM.

## Discussion

**Figure 9:**
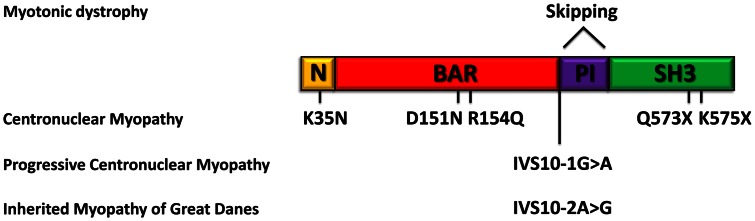
Schematic representation of the amphiphysin 2 domains and *BIN1* alterations in different myopathies. Schematic representation of the amphiphysin 2 protein domains and position of the known mutations causing autosomal recessive centronuclear myopathy, the new splice mutations resulting in rapidly progressive centronuclear myopathy and canine Inherited Myopathy of Great Danes. Myotonic dystrophy induces mis-splicing of *BIN1* exon 11. Nomenclature is based on isoform 1 (NM_139343).

In this study we identified and characterized *BIN1* mutations affecting the splicing of the muscle-specific exon 11, resulting in a rapidly progressing myopathy in humans and dogs. The IMGD dog is the first faithful mammalian model for *BIN1*-related centronuclear myopathy and particularly for the highly progressive form, and is the only characterized mammalian model available for preclinical trials of potential therapies for this severe congenital myopathy. Our data provide strong evidence for muscle-specific functions of amphiphysin 2 in membrane structural organization and remodelling and allow novel insights into the overlapping pathogenesis of centronuclear myopathy and myotonic dystrophy. A schematic representation of the amphiphysin 2 protein domains and of the position of the mutations and splicing alterations causing classical autosomal recessive centronuclear myopathy, rapidly progressive human CNM and canine IMGD as well as myotonic dystrophy is shown in Figure 9.

### *BIN1* mutations in classical and highly progressive centronuclear myopathies

Classical *BIN1*-related ARCNM has been described with neonatal or childhood onset, hypotonia and ptosis and all mutations were found in ubiquitously expressed exons [1], [13], [14], [15]. The muscle weakness was mildly to moderately progressive, and some patients could still walk at older age. In contrast, our patients with a splice mutation affecting the muscle-specific exon 11 showed a rapid disease progression involving strong care-dependence and leading to death within a few years, despite normal motor development and disease-onset not before 3.5 years. The histopathological findings of our patients and of the previously reported ARCNM cases partially overlap, including atrophy, prominent nuclear internalization and central dense areas upon NADH-TR staining of muscle sections. However, there is no evidence for type I fiber predominance in the muscle biopsies of our patients. Previous RT-PCR experiments demonstrated a progressive integration of exon 11 in *BIN1* mRNA during human skeletal muscle development [2]. We therefore hypothesize that the muscle-specific exon 11 plays a major role in muscle maintenance, rather than in early muscle development. This is in accordance with the highly progressive phenotype of humans and dogs with a disease onset several months or years after birth. Consistently, we detected amphiphysin 2 in muscle tissue, but RNA analysis revealed major skipping of *BIN1* exon 11. This suggests that the patients mainly express an embryonic *BIN1* isoform, which might not be able to assume the function of the adult *BIN1* isoform, possibly explaining the more progressive phenotype compared to patients with *BIN1* mutations in the ubiquitously expressed exons.

### The canine Inherited Myopathy of Great Danes is a faithful model for *BIN1*-related centronuclear myopathy

The characterization of the pathological mechanisms leading to *BIN1*-related CNM and the development of potential therapeutic approaches is obviated by the lack of a faithful animal model. *Bin1*-null mice are perinatally lethal [26], so that a comprehensive analysis of skeletal muscle alterations during disease development is not possible. We sought for dog breeds with molecularly unsolved congenital myopathies and we identified the canine Inherited Myopathy of Great Danes as a disease model reproducing the histological and physiological defects observed in *BIN1*-related CNM patients. IMGD has been reported for cases in Canada, Australia and UK and is characterized by generalized muscle atrophy, exercise intolerance, exercise-induced tremor and muscle wasting [29]. The disease typically starts before 10 months of age, is highly progressive, and most of the affected dogs are euthanized before 18 months of age due to severe debilitating muscle weakness. Histological examinations revealed internalized or central nuclei without evidence of inflammation, disruption of the sarcomeric architecture with central fiber areas devoid of myofibrils, and central accumulations of mitochondria and glycogen granules ([28], [29], [30] and our data). In addition, type I fiber predominance in combination with an increased expression of genes implicated in the slow-oxidative metabolism was described [35]. In this study we demonstrate that IMGD and progressive CNM have a comparable etiopathology and both conditions result from mutations of the AG acceptor splice site of the *BIN1* muscle-specific exon 11. The histopathology and the cellular organization defects of the human and canine muscle disorders are almost identical, we therefore consider IMGD as a faithful mammalian model for *BIN1*-related centronuclear myopathy.

### Veterinary implications

Some dogs of our IMGD cohort were found to be negative for *BIN1* mutations, suggesting that IMGD encompasses several disorders with similar clinical and overlapping histopathological features. The proven relationship of two affected Great Dane dogs demonstrates a common origin of the *BIN1* exon 11 splice mutation, and it is likely that all five affected dogs described here can be traced back to a common ancestor. As the muscle disorder is inherited as a recessive trait, and as canine pedigrees are generally highly inbred, it is likely that the mutation can be found in Great Dane dog populations from all over the world, as recently demonstrated for another autosomal recessive CNM form in Labrador retrievers [36]. It is therefore of veterinary medical interest to sequence *BIN1* exon 11 in Great Dane dogs. Also, veterinarians and veterinary pathologists should consider *BIN1* mutations as a possible cause of any unexplained progressive myopathy in dogs, especially when the biopsy displays internal nuclei and lobulated or indented sarcolemma.

### Insights into amphiphysin 2 muscle-specific functions and pathological mechanisms of centronuclear myopathy

Detailed immunohistochemical and ultrastructural analyses of muscles from patients and affected Great Dane dogs revealed common membrane alterations and abnormal accumulations of proteins regulating membrane trafficking. Similar findings were observed on biopsies from patients with *DNM2* or *MTM1* mutations [12], suggesting that mislocalization of triad proteins reflects common aberrations in CNM and that the amphiphysin 2 muscle-specific isoform plays an important role in triad formation and/or maintenance. This is in accordance with the known biochemical function of amphiphysin 2 and other N-BAR domain proteins to sense membrane curvature and to potentially induce curvature through the insertion of an amphipathic helix into the membrane bilayer. *In vitro* and cell culture experiments have led to the suggestion that the exon 11 encoded PI-binding motif is essential for membrane tubulation in cultured muscle cells [22]. Indeed, Drosophila mutated for amphiphysin, the ortholog of both amphiphysin 1 and amphiphysin 2, display an abnormal T-tubule system [24]. T-tubule alterations and muscle weakness were reproduced in murine *Tibialis anterior* injected with a U7 small nuclear RNA construct harboring an antisense sequence promoting *BIN1* exon 11 skipping [2]. However, nuclear centralization and atrophy were not observed, contrasting with the IMGD model. This difference might be species-related, is possibly due to a low efficacy of the AAV-U7 method or alternatively to the examination time point 4 months post injection. As the triad is the membrane structure controlling excitation-contraction coupling, this suggests that impaired excitation-contraction coupling and subsequent calcium homeostasis defects are a primary cause of the myopathy. Of note, abnormal intracellular calcium release was observed in isolated murine muscle fibers after *BIN1* shRNA-mediated knock-down [37]. Together with the present characterization of the IMGD model, these data indicate that amphiphysin 2 has an important muscle-specific role in triad structural maintenance, and provide additional evidence that triad modifications are a common defect in centronuclear myopathies, IMGD and myotonic dystrophies [2], [12].

Triads are not the only membrane compartment affected in patients and dogs harboring *BIN1* exon 11 mutations. We also noted central accumulations of caveolin 3 and dysferlin, two key regulators of membrane trafficking in skeletal muscle, numerous membranous whorls, and a peculiar remodeling of the sarcolemma, manifesting an indented fiber perimeter and invaginations towards the center of the fibers. Caveolin 3 regulates membrane tension at the sarcolemma and dysferlin controls membrane exocytosis in sarcolemmal membrane repair [33], [34]. As both proteins are also present on regenerating T-tubules [38], their mislocalization resulting from a *BIN1* mutation would be in accordance with defective T-tubule regeneration. Moreover, data mainly obtained in cultured cells support a key role of amphiphysins in the formation of endocytic vesicles [16], and a study in *Caenorhabditis elegans* suggested a role of amphiphysin in vesicle recycling [39]. Defects in intracellular signaling resulting from calcium defects and impaired transport of ion channels and growth factor might explain the muscle weakness and atrophy in *BIN1*-related CNM.

### Amphiphysin 2 links several forms of centronuclear myopathies and myotonic dystrophy

Our findings on the IMGD model uncovered possible links between *BIN1*-related and other forms of CNM. Altered triads and the presence of membranous whorls were reported for *MTM1* dog, mouse and zebrafish models as well as for patients with *MTM1* mutations involving protein loss [12], [31], [40], [41], [42]. Abnormal triad markers were also reported for *MTM1*-related and *DNM2*-related CNM [12], [43]. Dysferlin localization was not extensively studied in *MTM1*-CNM but was increased in the cytoplasm of a mouse model and in patients with *DNM2*-CNM [44]. Moreover, we found myotubularin localization was strongly impaired in IMGD muscles. These findings suggest that myotubularin and amphiphysin 2 are in the same pathway regulating membrane remodeling in skeletal muscle and strengthen the hypothesis of a common pathological mechanism of the X-linked and the autosomal recessive CNM forms.

Alternative splicing of *BIN1* exon 11 is mis-regulated in patients with myotonic dystrophy [2]. The parallel inclusion of exon 7 was noted, but its impact has not been assessed yet. Here we report the first mutation affecting the muscle-specific exon 11 of *BIN1* and having an impact on splicing. The major clinical and histological aspects of the patients and IMGD dogs include general muscle weakness, atrophy and nuclear centralization, consistent with the muscle phenotype in DM patients. Our data therefore support the hypothesis that mis-splicing of *BIN1* exon 11 partially accounts for the muscle-specific signs in myotonic dystrophy.

## Materials and Methods

### Ethics statement

Human sample collection was performed with informed consent from the patients according to the declaration of Helsinki and experimentation was performed as part of routine diagnosis. All dogs were examined with the consent of their owners. Blood and biopsies were obtained as part of routine clinical procedures for diagnostic purposes. Cheek cells were collected by owners or veterinarians using non-invasive swabs. As the data were from client-owned dogs undergoing normal veterinary exams, there was no “animal experiment” according to the legal definitions in Europe and the US. All local regulations related to clinical procedures were observed. Cryopreserved muscle specimens were processed and stored at the University of California, San Diego, under a tissue transfer approval from the institutional Animal Care and Use Committee.

### Molecular genetics

Human Genomic DNA was prepared from peripheral blood by routine procedures and sequenced for all coding exons and intron/exon boundaries of *MTM1*, *DNM2*, and *BIN1* as described elsewhere [1], [4], [5]. Patient 1 had a normal CTG repeat length at the DMPK locus (7 and 13 repeats) and was therefore excluded for myotonic dystrophy. Control DNAs were from healthy individuals of Turkish origin.

Dog DNA samples were extracted from cheek cells, venous blood or muscle biopsy specimens (cryosections or paraffin embedded tissue) by routine procedures and sequenced for all coding exons and intron/exon boundaries of canine *MTM1* [31], *PTPLA* [32] and *BIN1* (primer sequences in Table S1). Control samples were from a world-wide collection of healthy Great Danes as well as from healthy individuals of 13 other breeds.

### RNA studies

RNA was extracted from muscle biopsies by routine procedures and reverse transcribed using the SuperScript III kit (Invitrogen, Carlsbad, USA). Human and dog amplicons were cloned into the pGEM-T Easy vector (Promega, Madison, USA) and transfected into *E.coli* DH5α cells. Blue/white selection, repeated twice, resulted in 30 clones for the human cDNA and 3 clones for the canine cDNA. Control dog was an unaffected Drahthaar (German Wirehaired Pointer). Primer sequences are listed in Table S1.

### Protein studies

Western blot and immunofluorescence were performed using routine protocols. Biceps femoris and tibialis anterior biopsies from two affected dogs (14 months and 22 months, respectively) and from healthy age-matched Golden Retrievers or Belgian Shepherds as controls have been used for the analysis. Following antibodies were used for the study: R2406 (home-made rabbit anti-BIN1 PI binding domain), R2444 (home-made rabbit anti-BIN1 SH3 domain), R3062 (home-made rabbit anti-BIN1 exon 12 epitope), R2867 and R2868 (home-made rabbit anti-MTM1), mouse anti-GAPDH (Merck Millipore, Darmstadt, Germany), mouse anti-ryanodine receptor 1 (Affinity BioReagents, Golden, USA), mouse anti-SERCA 1 (Affinity BioReagents, Golden, USA), rabbit anti-dysferlin (Euromedex, Souffelweyersheim, France), goat anti-caveolin-3 (Tebu-BIO, Le-Perray-en-Yvelines, France), rabbit anti-caveolin-3 (Affinity BioReagents, Golden, USA), mouse anti-DHPR (Affinity BioReagents, Golden, USA), and mouse anti-dystrophin (Leica Microsystems, Germany). For immunohistofluorescence, transverse cryosections were prepared, fixed and stained by routine methods. Nuclei were stained with Hoechst or DAPI (Sigma-Aldrich, St. Louis, USA). Sections were mounted with slowfade antifade reagent (Invitrogen, Carlsbad, USA) and viewed using a laser scanning confocal microscope (TCS SP2; Leica Microsystems, Wetzlar, Germany) or a a Zeiss Axio Observer Z.1 microscope equipped with a 20×, 40× or 63× lens and Axioplan imaging with structured illumination (Carl Zeiss, Jena, Germany).

### Muscle histology

For histochemical analyses, transverse sections of muscle cryosections (8 µm) of vastus lateralis and biceps femoris muscle biopsies were stained with hematoxylin-eosin, modified Gomori trichrome, NADH-TR and myofibrillar ATPase and then assessed for centralized nuclei, fiber morphology, fiber type distribution, cores, protein accumulation and cellular infiltrations.

### Electron microscopy

Muscle biopsies were processed for electron microscopy as described previously [45]. Briefly, the tissue was fixed either in 6% phosphate-buffered glutaraldehyde (human patient) or in 2.5% paraformaldehyde, 2.5% glutaraldehyde, and 50 mM CaCl_2_ in 0.1 M cacodylate buffer at pH 7.4 (dog), and post-fixed with 2% OsO_4_, 0.8% K_3_Fe(CN)_6_ in 0.1 M cacodylate buffer (pH 7.4) for 2 h at 4°C and incubated with 5% uranyl acetate for 2 h at 4°C. Samples were dehydrated in graded series of ethanol and embedded in epoxy resin 812. Ultrathin sections (70 nm) were contrasted with uranyl acetate and lead citrate.

### Membrane tubulation assay

Murine C2C12 myoblasts were seeded on coverslips and transfected at 50–60% confluency using Lipofectamine 2000 (Invitrogen, Carlsbad, USA) either with GFP-BIN1 isoform 8 (including exon 11) or isoform 9 (excluding exon 11, both were a kind gift from Pietro de Camilli, Howard Hughes Medical Institute, USA). Cells were differentiated after 24 h by changing to medium containing 2% horse serum instead of FCS and fixed and stained after 5 days of differentiation by routine methods. Nuclei were stained with Hoechst/DAPI (Sigma-Aldrich, St. Louis, USA) and sections were mounted with slowfade antifade reagent and viewed using a laser scanning confocal microscope (TCS SP2; Leica Microsystems, Wetzlar, Germany).

### Web resources

1000 genomes - A Deep Catalog of Human Genetic Variation (URL: http://www.1000genomes.org/)

Database of Single Nucleotide Polymorphisms (dbSNP). Bethesda (MD): National Center for Biotechnology Information, National Library of Medicine. (dbSNP Build ID: 134).

(URL: http://www.ncbi.nlm.nih.gov/SNP/)

Exome Variant Server, NHLBI Exome Sequencing Project (ESP), Seattle, WA (URL: http://evs.gs.washington.edu/EVS/)

Online Mendelian Inheritance in Man (OMIM) (URL: http://www.omim.org/)

NNsplice: prediction of splice mutations (URL: http://www.fruitfly.org/seq_tools/splice.html)

Human Splicing finder (URL: http://www.umd.be/HSF/)

## Supporting Information

Figure S1 Western blot of canine muscle extracts using the anti-SH3 domain antibody. Compared to the control, the main skeletal muscle amphiphysin 2 isoform is strongly reduced in the IMGD dog. The protein levels of the other isoforms are also reduced, but still detectable.
(TIF)

Figure S2 Low-magnitude electron microscopy pictures of muscles from patient 1 and an affected dog demonstrate moderate Z-band streaming, mitochondrondrial accumulations and myofibrillar disarray.
(TIF)

Figure S3 Dog muscle sections labeled for developmental myosin. Signals were comparable in affected dog and control, suggesting that there is no excessive fiber regeneration.
(TIF)

Table S1 Primer sequences.
(XLSX)
