# Correction: Altered Splicing of the BIN1 Muscle-Specific Exon in Humans and Dogs with Highly Progressive Centronuclear Myopathy

**DOI:** 10.1371/annotation/22ca13f1-1ce9-4bb5-9c9e-98670f7c4240

**Published:** 2013-06-28

**Authors:** Johann Böhm, Nasim Vasli, Marie Maurer, Belinda S. Cowling, G. Diane Shelton, Wolfram Kress, Anne Toussaint, Ivana Prokic, Ulrike Schara, Thomas James Anderson, Joachim Weis, Laurent Tiret, Jocelyn Laporte

The middle initial of fourth author's name was missing. The correct name is Belinda S Cowling. 

