# Supplementary figures and images for: Altered Splicing of the BIN1 Muscle-Specific Exon in Humans and Dogs with Highly Progressive Centronuclear Myopathy
Source: PLoS Genet. 2013 Jun 6;9(6):e1003430. doi: 10.1371/journal.pgen.1003430 (PMC3675003; doi:10.1371/journal.pgen.1003430)

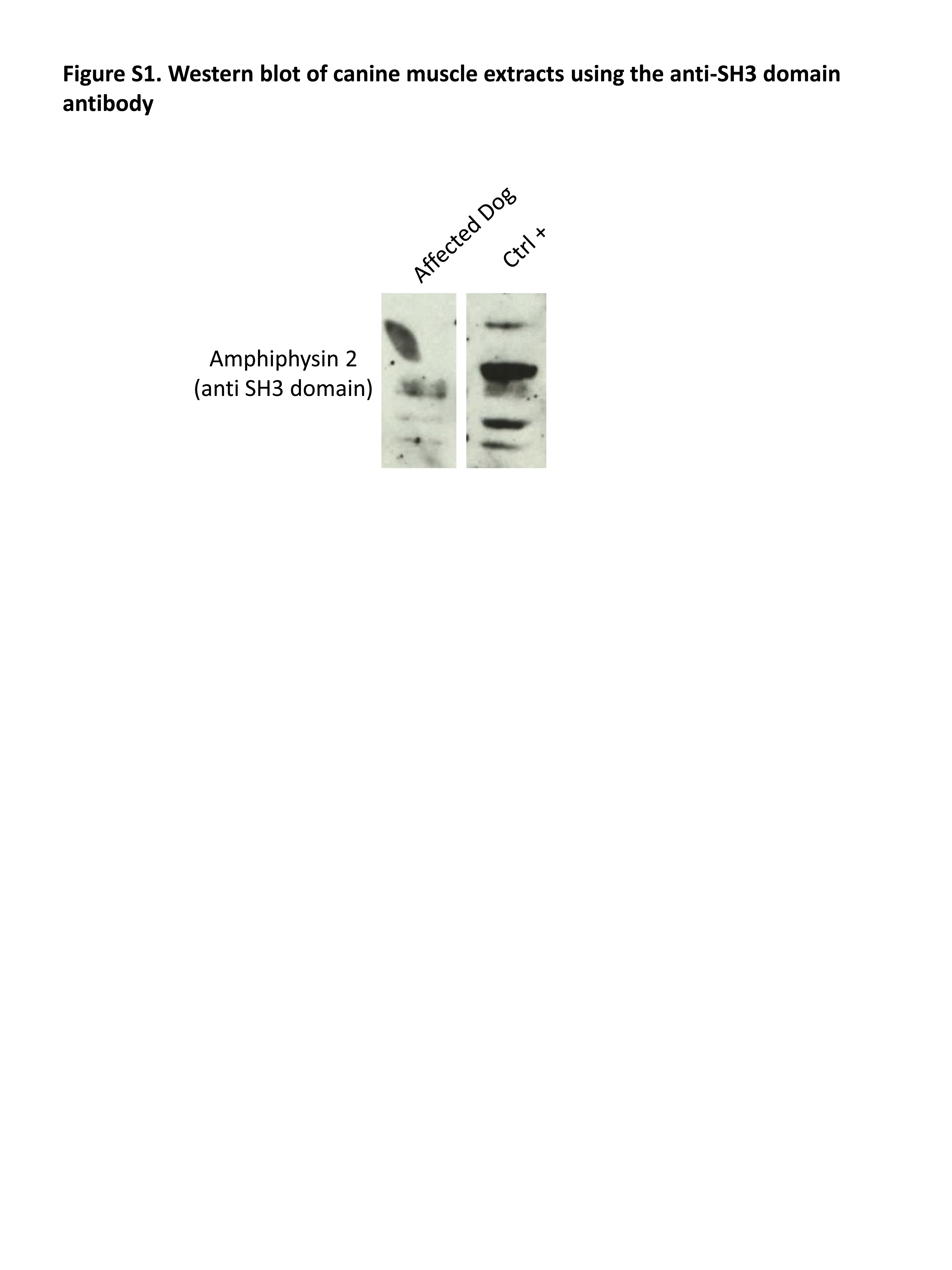

Supplement: Figure S1 — Western blot of canine muscle extracts using the anti-SH3 domain antibody. Compared to the control, the main skeletal muscle amphiphysin 2 isoform is strongly reduced in the IMGD dog. The protein levels of the other isoforms are also reduced, but still detectable. (TIF) [file pgen.1003430.s001.tif]

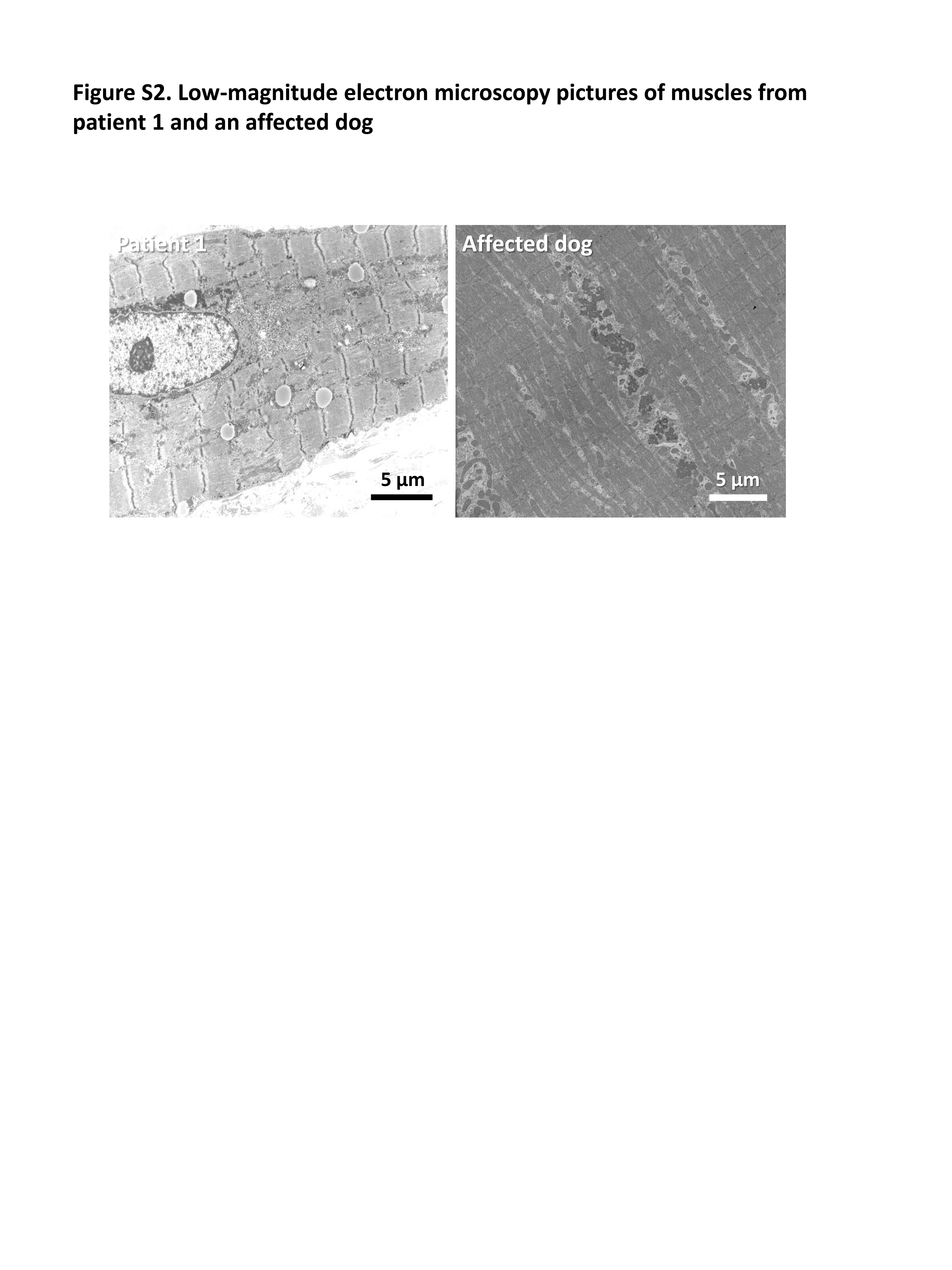

Supplement: Figure S2 — Low-magnitude electron microscopy pictures of muscles from patient 1 and an affected dog demonstrate moderate Z-band streaming, mitochondrondrial accumulations and myofibrillar disarray. (TIF) [file pgen.1003430.s002.tif]

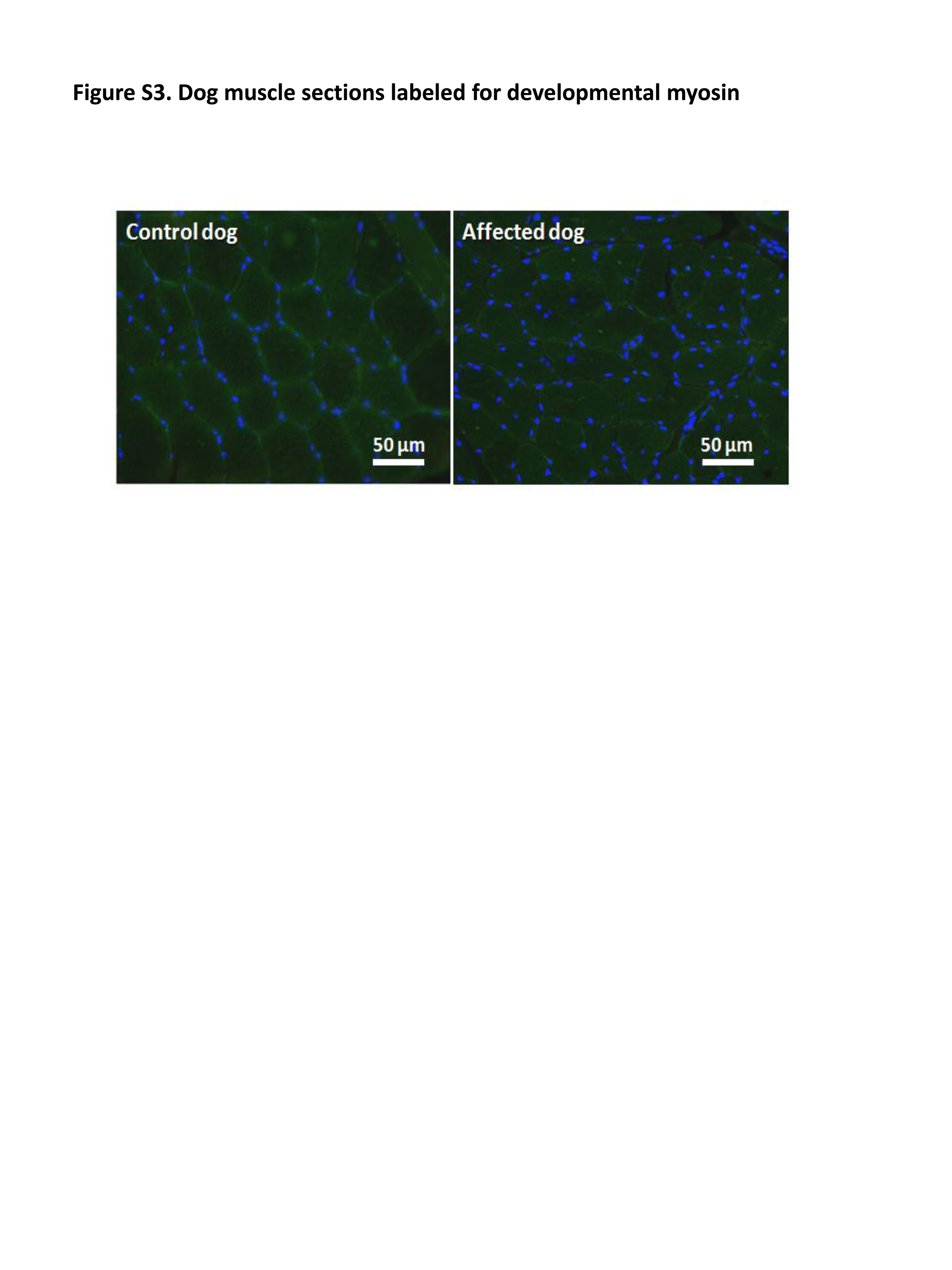

Supplement: Figure S3 — Dog muscle sections labeled for developmental myosin. Signals were comparable in affected dog and control, suggesting that there is no excessive fiber regeneration. (TIF) [file pgen.1003430.s003.tif]
